# Fast camera spatial characterization of photonic polarization entanglement

**DOI:** 10.1038/s41598-020-62020-z

**Published:** 2020-04-10

**Authors:** Christopher Ianzano, Peter Svihra, Mael Flament, Andrew Hardy, Guodong Cui, Andrei Nomerotski, Eden Figueroa

**Affiliations:** 1 Department of Physics and Astronomy, Stony Brook University, Stony Brook, NY 11794 USA; 2 Department of Physics, Faculty of Nuclear Sciences and Physical Engineering, Czech Technical University, Prague, 115 19 Czech Republic; 3 Department of Physics and Astronomy, School of Natural Sciences, University of Manchester, Manchester, M139PL UK; 4 Brookhaven National Laboratory, Upton, NY 11973 USA; 5 Department of Physics, University of Toronto, Toronto, ON M5S 3H7 Canada; 6 Brookhaven National Laboratory, Upton, NY 11973 USA

**Keywords:** Quantum optics, Quantum information

## Abstract

Scalable technologies to characterize the performance of quantum devices are crucial to creating large quantum networks and quantum processing units. Chief among the resources of quantum information processing is entanglement. Here we describe the full temporal and spatial characterization of polarization-entangled photons produced by Spontaneous Parametric Down Conversions using an intensified high-speed optical camera, Tpx3Cam. This novel technique allows for precise determination of Bell inequality parameters with minimal technical overhead, and for new characterization methods for the spatial distribution of entangled quantum information. The fast-optical camera could lead to multiple applications in Quantum Information Science, opening new perspectives for the scalability of quantum experiments.

## Introduction

Ever since the original experiments with entangled photons^1^, photonic entanglement has become a remarkable resource in the development of quantum technologies, including entanglement over long-distance for quantum communication^2^, entanglement swapping^3^, teleportation between a photon and an atomic ensemble^4^, violation of the CHSH (Clauser-Horne-Shimony-Holt) inequality measured over long distances^5,6^ and entanglement of spin waves among four quantum memories^7^.

The creation of quantum networks of many such quantum devices in which entanglement is shared among multiple network nodes is the next technological frontier for the successful development of these applications. Easy-to-use, scalable, and compact characterization devices, providing all the information regarding entanglement in near-real-time are fundamental for further large-scale network developments.

Recent developments have shown that spatial characterization of entangled states with single-photon sensitive cameras provides access to a myriad of new possibilities, such as imaging high-dimensional entanglement^8^, generalized Bell inequalities^9^ and the study of Einstein-Podolsky-Rosen non-localities^10,11^. However, most of these measurements used resource-intensive methods, such as sequential scanning or multiple stand-alone detectors. More recent experiments used low noise CCD^12,13^ and electron-multiplying CCD (EMCCD)^14^ cameras to study spatial quantum correlations between twin beams of pulsed parametric down-conversion photon sources. In those cases, the temporal resolution was determined by the pulsed nature of the source, albeit the cameras were not operated in the single-photon regime, as the results required integrating millions of photons per laser pulse. EMCCDs were also used in the single-photon mode to study spatial correlations using an SPDC source^15^. However, the exposure time was 33 ms, so albeit the individual single photons were indeed spatially resolved, multiple photon pairs were registered in a single shot. The main motivation in the above studies was a demonstration of sub-shot noise that can be achieved by exploiting quantum correlations. These experiments did not perform entanglement characterization, as it would require to analyze the pair coincidences.

Early studies of entanglement with modern imagers used an EMCCD camera with an active area of 201 × 201 pixels and frame readout-rate of 5 Hz^8^. Although the EMCCD quantum efficiency was up to 90%, a long exposure time of about 1 ms was necessary to operate this device at minimum photon rates to avoid multiple photons in the same frame. Furthermore, to achieve single-photon level sensitivity the EMCCD camera operated at a low temperature of  − 85 °C, which is typical for this type of cameras.

Progress on quantum imaging with cameras was achieved using intensified CMOS and CCD cameras^16–21^. Flexible readout architectures allow kHz continuous framing rates in CMOS cameras. Additionally, nanosecond scale time resolution for single photons can be achieved by gating image intensifiers in the cameras. For example, an intensified sCMOS camera was used to observe Hong-Ou-Mandel interference^22^, where the entangled photons were collected on a 700 × 22 pixel area at a framing rate of 7 kHz, and their coincidence was ensured using a narrow 40 ns intensifier gate. Photon acquisition statistics can also be enhanced by using multiple triggers during a single frame, so the camera integrates multiple photons within a single acquired image. This approach was employed in^19^, where the idler photon from an entangled pair was used to gate an intensified CCD camera. Although many thousands of photons were imaged in a single frame of the camera, allowing the spatial characterization of the photon’s angular momentum, the framing-rate was only 4 Hz, and the spatial information of the idler photon was not available since it was used for triggering.

This low throughput remains a severe limitation to resolve spatial characterization of entanglement in real-time. Here we show how development from the high-energy physics community, the intensified Tpx3Cam camera^23^ can be converted into a quantum characterization device of photonic polarization entanglement. This setup allows for imaging and time-stamping of a continuous stream of entangled photons with an excellent spatial and temporal resolution (55 × 55 μm^2^, 1.5 ns), providing a high signal-to-background ratio. We emphasize that the Tpx3Cam readout is data-driven, with a high throughput of  ≈ 10 M photons per second, which is several orders of magnitude higher than the conventional cameras discussed above. The main advantage of the camera is that it simultaneously gives access to the spatial and temporal information for each recorded photon and, hence, allows to study, at the same time, the spatial and temporal correlations of multiple photons (e.g. pairs, triplets), which was not the case for the experiments mentioned above. In the following, we demonstrate this for the case of spatial characterization of photonic polarization entanglement, which makes use of spatially resolved coincidences of photon pairs.

## Experimental Setup

**Figure 1:**
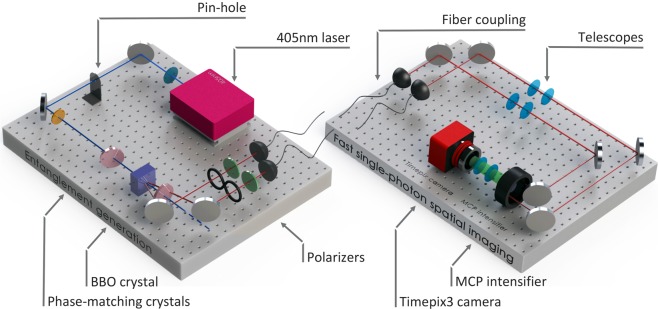
Experimental layout for entanglement generation (1; left) and characterization (2; right): (1) The source utilizes a blue pump laser diode tuned to a wavelength of *λ* = 405 nm and a pair of Type I BBO crystals with optical axes perpendicular to one another to generate signal and idler photons entangled in polarization at a wavelength of *λ* = 810 nm. The entangled photons undergo individual transformations using polarizers to evaluate Bell’s inequality parameters and are then fiber coupled. (2) Exiting from the fibers photons are mode-matched and detected by an image intensifier before registration with the Tpx3Cam camera.

We study the characterization of SPDC-based quantum polarization entanglement using fast 2D imaging with a Tpx3Cam. The experimental layout is shown in Fig. 1.

### Entangled-photon source

Our entanglement source (QuTools QuED^24^) utilizes a blue pump laser diode tuned to wavelength *λ* = 405 nm, and a pair of Type *I* non-collinear BBO crystals with optical axes perpendicular to one another to generate signal and idler photons at wavelength *λ* = 810 nm. The first crystal optical axis and the pump beam define the vertical plane. Owing to Type *I* phase matching, an incoming photon which is vertically polarized gets down-converted and produces two horizontally polarized photons in the first crystal, whereas a horizontally-polarized photon going through these crystals would get down-converted in the second crystal producing two vertical photons. An additional pair of birefringent crystals ensures maximum spatial overlap of the down-converted photons by pre- and post-compensating for differences in effective optical path lengths of signal and idler. The produced state has the form:

1 $$|{\phi }^{\pm }\rangle =\frac{|HH\rangle \pm |VV\rangle }{\sqrt{2}}$$

Signal and idler photons are spatially separated and collected using single-mode, polarization non-maintaining fibers, with a linear polarizer, used for projective measurements, before each coupler.

### Intensified fast-camera: Tpx3Cam

An intensified camera, Tpx3Cam^23^, achieves imaging with single-photon sensitivity when coupled to an image intensifier and allows time-stamping of incident photons with 1.5 ns time granulation. The Tpx3Cam consists of a light-sensitive silicon sensor bump-bonded to Timepix3, a time-stamping readout chip^25^. The sensor-chip assembly has 256 × 256 pixels of 55 × 55 μ*m*^2^ each. The silicon sensor in the camera has a thin entrance window with an anti-reflective coating providing excellent quantum efficiency^26^. The sensor is optimized for emission spectrum of the *P*47 scintillator^27^. The non-intensified version of Tpx3Cam has been used before for the velocity mapped ion imaging^28^ while the intensified version of its predecessor, TimepixCam, has previously been used for fluorescent lifetime imaging, which required single-photon sensitivity, similar to this application^29^. This is the first time when Tpx3Cam is employed in the single-photon regime.

The Timepix3 processing electronics in each pixel records the time-of-arrival (TOA) of hits that cross a preset threshold and stores it as a time-code in a memory inside the pixel. The time-over-threshold (TOT) is also recorded, serving as an estimate of the light flux seen by the pixel. The individual pixel dead time is caused by digitization and TOT of measured signal and is of the order of 1 μs. The hit pixel does not affect the performance of other pixels since all pixels in the chip function independently. The Timepix3 readout is data-driven, and only the pixels with signals above the threshold are recorded. The camera can operate continuously and does not require a trigger as the pixels transfer the data asynchronously within microseconds after being hit. The maximum camera throughput is 80 Mpix/s^23,30^. SPIDR can also accept and time stamp an external trigger pulse, independent of the Timepix3 connection.

The intensifier in front of the camera is an off-the-shelf vacuum device^31^, which consists of a photocathode followed by a chevron micro-channel plate (MCP) and fast *P*47 scintillator with a signal rise time of  ~7ns^32^. Photons are first converted to photoelectrons in the photocathode and then amplified in the MCP before producing a flash of light in the scintillator. The 18 mm diameter scintillator screen is projected on to the 14 × 14 mm^2^ sensor with a relay lens with no magnification^33^. The quantum efficiency of the photocathode used for the experiments attains  ≈ 18% at a wavelength of 810 nm.

The camera was calibrated to equalize the response of all pixels by adjusting the individual pixel thresholds^34^. After this procedure, the effective threshold to fast light flashes from the intensifier is 600–800 photons per pixel depending on the wavelength. A small (≈0.1%) number of hot pixels was masked to prevent logging high rates of meaningless data.

## Experimental Results

### Benchmarking: entanglement characterization

Our procedure starts by evaluating the entangled state produced by the source. We assume the state to be in a superposition of two Bell-states of the form:

2 $$\begin{array}{ccc}\left|\psi \right\rangle =\cos \theta \left|{\phi }^{+}\right\rangle +{e}^{i\delta }\sin \theta \left|{\phi }^{-}\right\rangle =\frac{\left(\cos \theta +{e}^{i\delta }\sin \theta \right)}{\sqrt{2}}\left|HH\right\rangle +\frac{(\cos \theta -{e}^{i\delta }\sin \theta )}{\sqrt{2}}\left|VV\right\rangle  &  & \end{array}$$

Using a density matrix $\rho =\left|\psi \right\rangle \left\langle \psi \right|$, after projection of the two photons by polarizers with angles *α* and *β*, we obtain an expectation value for the measurements of coincidences:

3 $$\begin{array}{ccc}{P}_{VV}(\alpha ,\beta )=\,{\rm{Tr}}\,\left\{\rho {\widehat{M}}_{\alpha \beta }\right\}={c}_{0}+{c}_{1}\cos 2\beta +{c}_{2}\sin 2\beta  &  & \end{array}$$

here, the operator ${\widehat{M}}_{\alpha \beta }=\left|{V}_{\alpha }{V}_{\beta }\right\rangle \left\langle {V}_{\alpha }{V}_{\beta }\right|$ denotes the projection onto a vertical polarization state. In the basis of BBO crystal we have:

4 $$\begin{array}{ccc}|{V}_{\alpha }{V}_{\beta }\rangle =\sin \alpha \sin \beta |HH\rangle -\sin \alpha \cos \beta |HV\rangle -\cos \alpha \sin \beta |VH\rangle +\cos \alpha \cos \beta |VV\rangle  &  & \end{array}$$

with $${c}_{0}=\frac{1-\sin 2\theta \cos \delta \cos 2\alpha }{4},{c}_{1}=\frac{\cos (2\alpha )-\cos \delta \sin 2\theta }{4}$$ and $${c}_{2}=\frac{\cos 2\theta \sin 2\alpha }{4}$$.

The incoming photon pairs from BBO crystal and background are indicated as *N*_0_ and *N*_*d*_ respectively. Then the total coincidence can be fitted as the equation:

5 $$\begin{array}{ccc}N(\alpha ,\beta )={N}_{0}{P}_{VV}(\alpha ,\beta )+{N}_{d}={C}_{0}+{C}_{1}\cos (2\beta )+{C}_{2}\sin (2\beta ) &  & \end{array}$$

where $${C}_{0}=-\frac{{N}_{0}\cos \delta \sin 2\theta }{4}\cos 2\alpha +\frac{{N}_{0}+4{N}_{d}}{4},{C}_{1}=\frac{{N}_{0}}{4}\cos 2\alpha -\frac{{N}_{0}\cos \delta \sin 2\theta }{4}$$ and $${C}_{2}=\frac{{N}_{0}\cos 2\theta }{4}\sin 2\alpha $$.

**Figure 2:**
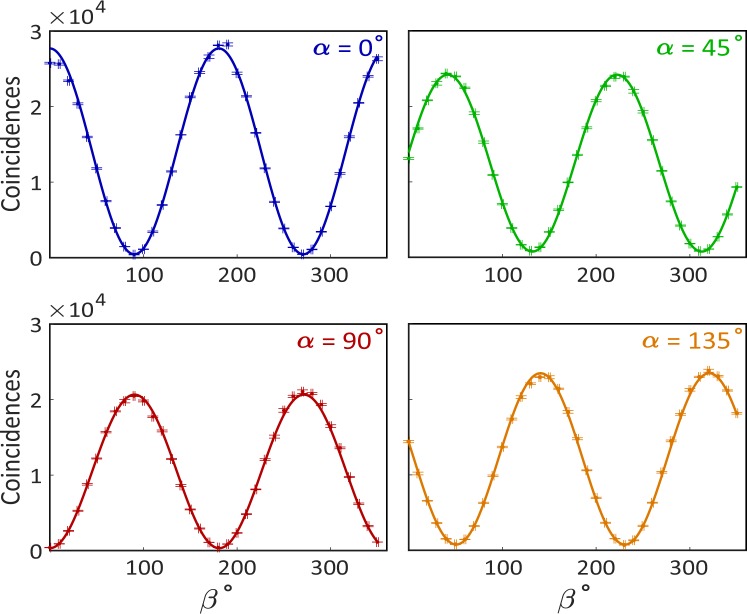
Coincidence for all four linear polarizer angles *α* = 0° (blue), 45° (green), 90°(red), 135°(orange) using quTools^47^. Polarizer angle *β* was varied over full 360° at a step of 10° for each of four *α*’s. Five data points were taken and averaged at each polarizer combination. The uncertainty of polarizer angles is 1°. Curves are fitted with sine functions predicted from pure state model as discussed in entanglement characterization. The pure quantum state is fitted to be $\left|\psi \right\rangle =0.989\left|{\phi }^{+}\right\rangle +(0.076-0.130i)\left|{\phi }^{-}\right\rangle$.

Experimentally, we evaluate the rate of coincidences using two single-photon counting modules, as a dependence on the polarization angles *α* and *β*, which are set by rotating two polarizers (cf. Fig. 1). The coincidence data for different settings of the polarizers and the respective fitting curves are shown in Fig. 2, where we see the oscillation predicted by the simple theory described above. We numerically fit the parameters *N*_0_, *θ*, *δ* and *N*_*d*_ to the experimental data, obtaining the following results: *N*_0_ ± Δ*N*_0_ = 47640 ± 2800, *N*_*d*_ ± Δ*N*_*d*_ = 380 ± 830, *θ* ± Δ*θ* = −0.15 ± 0.10 and *δ* ± Δ*δ* = 2.10 ± 0.48. Hence, the produced entangled state is: $\left|\psi \right\rangle =0.989\left|{\phi }^{+}\right\rangle +(0.076-0.130i)\left|{\phi }^{-}\right\rangle$.

### Benchmarking: Bell inequality violation using SPCM

Our next step is to calculate the Clauser-Horne-Shimony-Holt (CHSH) inequality violation^35,36^ using SPCM (Single Photon Counting Module). The inequality can be written as:

6 $$\begin{array}{ccc}S=E(\alpha ,\beta )+E(\alpha {\prime} ,\beta )-E(\alpha ,\beta {\prime} )+E(\alpha {\prime} ,\beta {\prime} )\le 2 &  & \end{array}$$

where $E(\alpha ,\beta )=\frac{{N}_{VV}(\alpha ,\beta )+{N}_{HH}(\alpha ,\beta )-{N}_{VH}(\alpha ,\beta )-{N}_{HV}(\alpha ,\beta )}{{N}_{VV}(\alpha ,\beta )+{N}_{HH}(\alpha ,\beta )+{N}_{VH}(\alpha ,\beta )+{N}_{HV}(\alpha ,\beta )}$ from the fitted curves in Fig. 2. *N*_*xy*_(*α*, *β*) with *x*, *y* = *V*, *H* are defined to represent counting schemes for linear polarizer angles such that *N*_*VV*_(*α*, *β*) = *N*(*α*, *β*), *N*_*HV*_(*α*, *β*) = *N*(*α* + 90°, *β*), *N*_*VH*_(*α*, *β*) = *N*(*α*, *β* + 90°), and *N*_*HH*_(*α*, *β*) = *N*(*α* + 90°, *β* + 90°). We obtain the values shown in Table 1.

**Table 1:** Number of coincidences used to calculate the S-value.

(*α*, *β*)°	N_V V_	N_HV_	N_V H_	N_HH_	E(α, β)
(0,22.5)	17656	4393	3344	23767	0.685243
(0,67.5)	3344	23767	17656	4393	−0.685243
(45,22.5)	19064	2984	5516	21596	0.654174
(45,67.5)	21596	5516	2984	19064	0.654174

Using these values, we calculate the *S* parameter *S* = 2.679 ± 0.007 > 2, demonstrating the violation of the CHSH inequality. The uncertainty is calculated using $\Delta S=\sqrt{{\sum }_{\alpha ,\beta }\Delta E{(\alpha ,\beta )}^{2}}$ and

7 $$\begin{array}{ccc}\Delta E & = & \frac{2[{N}_{VV}(\alpha ,\beta )+{N}_{HH}(\alpha ,\beta )][{N}_{HV}(\alpha ,\beta )+{N}_{HV}(\alpha ,\beta )]}{{\left({N}_{VV}(\alpha ,\beta )+{N}_{HH}(\alpha ,\beta )+{N}_{HV}(\alpha ,\beta )+{N}_{HV}(\alpha ,\beta )\right)}^{2}}\\  & \times  & \sqrt{\frac{1}{{N}_{VV}(\alpha ,\beta )+{N}_{HH}(\alpha ,\beta )}+\frac{1}{{N}_{HV}(\alpha ,\beta )+{N}_{VH}(\alpha ,\beta )}}\end{array}$$

## Entanglement Characterization with Tpx3Cam

Having set a benchmark for the measurements, we proceed to characterize the same entanglement source using the Tpx3Cam. Instead of being measured in the standalone single-photon detectors, the entangled pairs are sent to another experimental setup where they are converted to photoelectrons, amplified in different regions of the intensifier and sub-sequentially time-stamped in the fast camera.

**Figure 3:**
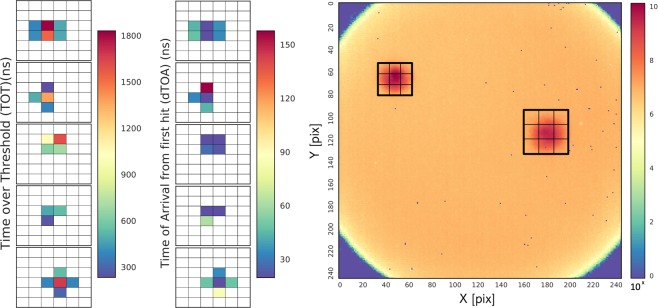
(**a**) Examples of zoomed-in photon hits, the left column shows TOT in ns; the right column shows relative TOA from the first hit pixel in ns; and (**b**) 2*D* occupancy map of the sensor (256 × 256 pixels) showing the photon hits for the full statistics of a five-minute run, the color encodes the number of times a particular pixel was hit in decimal log scale.

The photon pairs were recorded continuously by the camera for a given period of 5 min, for each combination of polarizations. Figure 3a) shows five examples of individual single-photon events in the camera with their time (TOA, right column) and amplitude (TOT, left column) information using raw data. Note that there is an anti-correlation of TOA and TOT for the same pixels, which can be used to improve the time resolution, as discussed later. The two beam spots coming from the optical fibers are visible in Fig. 3b), corresponding to the areas of highest occupancy. As expected, the intensity distributions in the fibers follow the Gaussian modes. The rate of photons in these regions was  ≈ 20 kHz, determined primarily by the output rate of the photon source at the fiber end (typically  ≈ 100 kHz) and the intensifier quantum efficiency.

The background, uniformly distributed over the photocathode surface in the occupancy map in Fig. 3b), is caused by spurious dark counts from the photocathode. This rate is  ≈ 50 times smaller than the measured single-photon rate in the fiber areas and could be further reduced by cooling the intensifier, which in our measurements was operated at room temperature. We also note that the background photoelectrons arrive at random times and thus suppressed by requiring coincidence between the two photons, as shown below.

### Data processing

To perform a Bell’s inequality measurement using data from the fast camera, we gathered 72 five-minute long datasets corresponding to different settings of the polarizers. The raw data is processed following several steps: *i*) time-ordering of the hit pixels, *ii*) identification of the pixel clusters corresponding to the single-photon hits, *iii*) centroiding of the pixel clusters, *iv*) TOT corrections to improve the time resolution, *v*) calculating the number of coincidences and *vi*) Bell analysis.

#### I. Time-ordering

Tpx3Cam reads out the hit pixels asynchronously, which might alter the time order, especially at high rates. Thus, the first step of the data processing is to time-order the pixels to prepare the data for the cluster finding.

#### II. Clustering

Clusters are groups of pixels adjacent to each other and within a preset time window. We used a recursive algorithm to look for the clusters: for a pixel, a 1 μs time window is applied to select other pixels close in space and time to the first one. Each pixel in a cluster should have a neighboring pixel separated not more than 300 ns apart. The algorithm then chooses another pixel, not contained in a cluster, shifting the time window and starting the process anew.

#### III. Centroid

A photon hit in the camera comprises, on average,  ≈ 4 pixels with measured TOA and TOT, which allows applying a centroiding algorithm. The TOT information is used as a weighting factor, helping to define the geometrical center of the cluster, yielding an estimate of the coordinates *x*, *y* of the incoming single-photon. The arrival time of the photon is estimated by using TOA of the pixel with the largest TOT in the cluster, correspondingly called TOA_centroid_ and TOT_centroid_.

#### IV. TOT correction

Photons in the entangled pairs are simultaneous. Therefore, they will have the same time-stamps within the time resolution. Precise timing is a powerful handle to reduce the random background, thus improving the camera time resolution and the signal-to-background ratio. For this, the timing information must be corrected to account for the so-called time walk. In the Timepix3 front-end electronics, within each pixel, the discriminator keeps a constant threshold, so larger signals cross the threshold earlier, producing smaller TOA and larger TOT values. The correlation of TOA and TOT allows to calibrate the constant threshold effect and, therefore, improves the timing resolution.

Typically, the correction requires a time reference, for example, from a laser, to determine the shift, as in the ion imaging experiments^28^. Since, in these experiments, the entangled pairs are generated continuously, we had to develop a different procedure that does not rely on an external time reference. TOA_centroid_ was used as zero time reference for the cluster, giving a time difference defined by:

8 $${\rm{dTOA}}({\rm{TOT}},{{\rm{TOT}}}_{{\rm{centroid}}})={\rm{TOA}}({\rm{TOT}})-{{\rm{TOA}}}_{{\rm{centroid}}}({{\rm{TOT}}}_{{\rm{centroid}}})$$

This can be calculated for each pixel within the cluster and associated with the pixel TOT and cluster TOT_centroid_ value. Combining the data from all runs a trend was observed that the value of dTOA(TOT,TOT_centroid_) is constant for large TOT_centroid_ values (typically for TOT_centroid_ > 1500 ns), removing dependency on TOT_centroid_. Thus a universal lookup table of dTOA(TOT) can be obtained. This procedure reduces the time difference between entries within a given cluster from  ~ 100 ns down to a few ns and can be applied to TOA_centroid_, improving the timing resolution.

**Figure 4:**
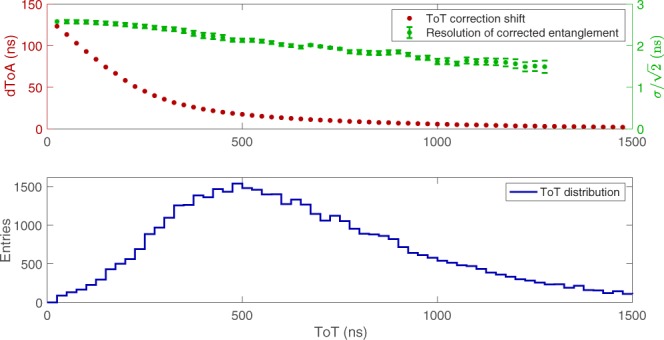
Time correction and time resolution as a function of TOT (top) and TOT distribution (bottom). In the top figure, the red dots show the TOA shift due to the TOT correction. The green dots show the time resolution as a function of TOT.

The time resolution after the TOT correction is shown in the top graph of Fig. 4 as a function of TOT. The time resolution is determined from the distribution of time difference between two entangled photons. The distribution is fit with a Gaussian function, and the time resolution per photon is defined as the sigma of the fit divided by $\sqrt{2}$ assuming equal resolution for each of the two photons. To determine the dependence of the resolution on TOT, we required that both photons have TOT greater or equal than a specific value on the TOT axis. The distribution of the TOT values is shown in the bottom graph of Fig. 4.

#### V. Time coincidences

**Figure 5:**
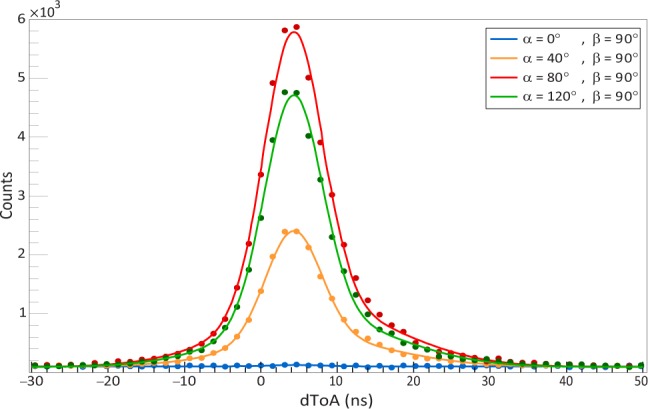
Distribution of time difference between two photons in different fibers for selected pairs of polarization settings combinations: *α* = 0° (blue), *α* = 40° (orange), *α* = 80° (red) and *α* = 120° (green) with *β* = 90°. The amplitudes for different polarization combinations are determined by the entangled state projection. Flat background results from uncorrelated photon background. The total number of coincidences is calculated by integration over the Gaussian curves.

To identify pairs of simultaneous photons, we selected areas of the sensor corresponding to regions illuminated by the fibers. The corresponding square areas were 42 × 42 pixels for the right fiber and 30 × 30 pixels for the left fiber, as shown in Fig. 3b). Then, for each photon detected in one region, we looked for its associated pair at the closest time in the second region. The time difference distribution for these detected pairs is shown in Fig. 5 for several settings of the polarizers, as defined in the Bell measurement above. The prominent peaks correspond to the pairs of entangled photons, while the small flat background corresponds to random coincidences of uncorrelated photons. Due to the finite quantum efficiency and other losses, not each photon from the source will have a detected synchronous partner in the other fiber. In this case, it will be paired with a random photon, either from the photon source (more likely) or the spurious photocathode counts, giving rise to the flat background.

Each distribution was fit to a function consisting of two Gaussians and a constant accounting for the flat background of random coincidences. The number of coincidences was estimated as the area under the Gaussian functions. The dependence of the number of coincidences on the polarizer settings indicates that the operation of the camera detection setup closely resembles the SPCM operation, despite the use of an entirely different registration scheme for single photons.

### Bell’s inequality violation with a fast camera

**Figure 6:**
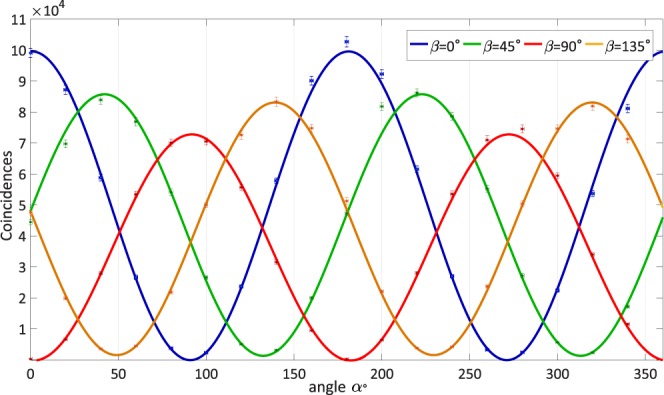
Coincidences as a function of polarization: showing the dependence of the signal amplitude (number of coincidences) for different settings of a Clauser-Horne-Shimony-Holt type Bell inequality test. We use the same color conventions as Fig. 2, with the fix polarization *β* = 0° (blue), *β* = 45°(green), *β* = 90°(red) and *β* = 135°(orange). The uncertainty in polarization is 1°. Fitting parameters are listed in Table 2.

The next step consists in studying the dependence of the coincidence measurements on the polarization projective measurements of the two-photon state. In the measurements, one polarizer was varied in 20° steps for four fixed values of the other polarizer: 0°, 45°, 90° and 135°. The dependence of the number of coincidences versus the polarizer angle is shown in Fig. 6. The data points were fitted with a sine function with the period, phase, amplitude and offset as free parameters. The fit results are shown in Table 2.

**Table 2:** Parameters of the sin functions used to fit the polarization function. The function $A\sin \left(\frac{2\pi }{T}(x+\phi )\right)+B$ is used to fit the values. Amplitude is defined as *A*, period is *T*, phase shift is *ϕ*, and offset is *B*.

Polarization *β*	Amplitude *A*	Period *T* [deg]	Phase shift *ϕ* [deg]	Offset *B*
0	1.000	180.2 ± 0.2	−44.0 ± 0.2	0.999 ± 0.005
45	0.851 ± 0.005	180.8 ± 0.2	−3.8 ± 0.2	0.877 ± 0.004
90	0.735 ± 0.004	180.5 ± 0.2	46.5 ± 0.2	0.732 ± 0.004
235	0.822 ± 0.005	180.2 ± 0.2	94.2 ± 0.2	0.853 ± 0.004

With these experimental parameters, we follow the procedure outlined in the benchmarking section, to obtain a Bell-state inequality S-value with the Tpx3Cam setup. The obtained value is 2.78 ± 0.02, well above the classical limit of 2 and closer to the Tsirelson Bound of $2\sqrt{2}$ (2.82) than with the SPCM measurements. We attribute this improvement to the better rejection of random background enabled by the fast camera. In these measurements, the signal is estimated with a fitting procedure described in the previous section. This procedure automatically accounts for the background and camera time resolution using the same dataset and gives a better estimate of the signal. We verified that the fast camera data presented in Fig. 6 have better visibility than the standard analysis data in Fig. 2, which should lead to higher S-values.

### Position dependent bell analysis

**Figure 7:**
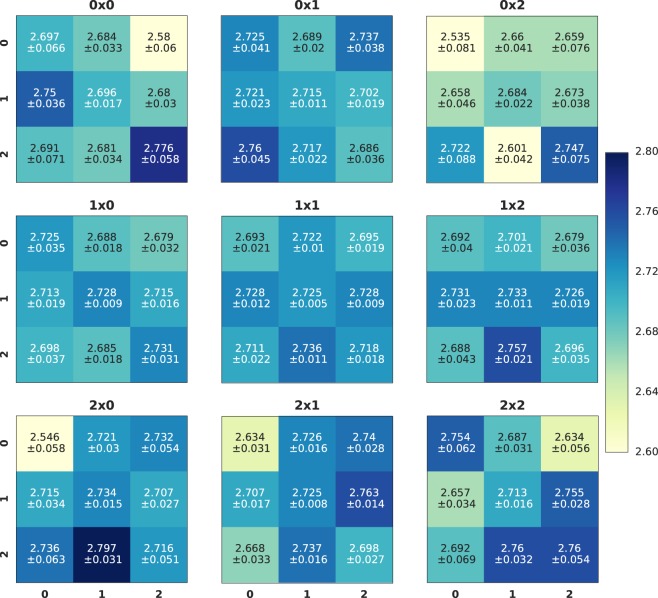
Table of *S*-values for sub-area matrices. In this configuration, the two areas on the fast camera that are illuminated by photons from the fibers are divided into subareas, forming two 3 × 3 matrices. The coincidence thus decomposes into that of 81 possible pairs of a combination of subareas. Using these coincidences, we calculated the CHSH inequality violation and plotted the resulting *S*-values in the form of 81 blocks in nine 3 × 3 matrices. The *S*-values are color-coded with the corresponding uncertainty shown in the center of the box. This produces an intuitive illustration of the spatial distribution of entangled photon pairs. The digits above each matrix give the position of the photon in the first fiber: 0 × 0 corresponds to the top left corner, 0 × 2 to the top right corner, 1 × 1 to the center.

**Figure 8:**
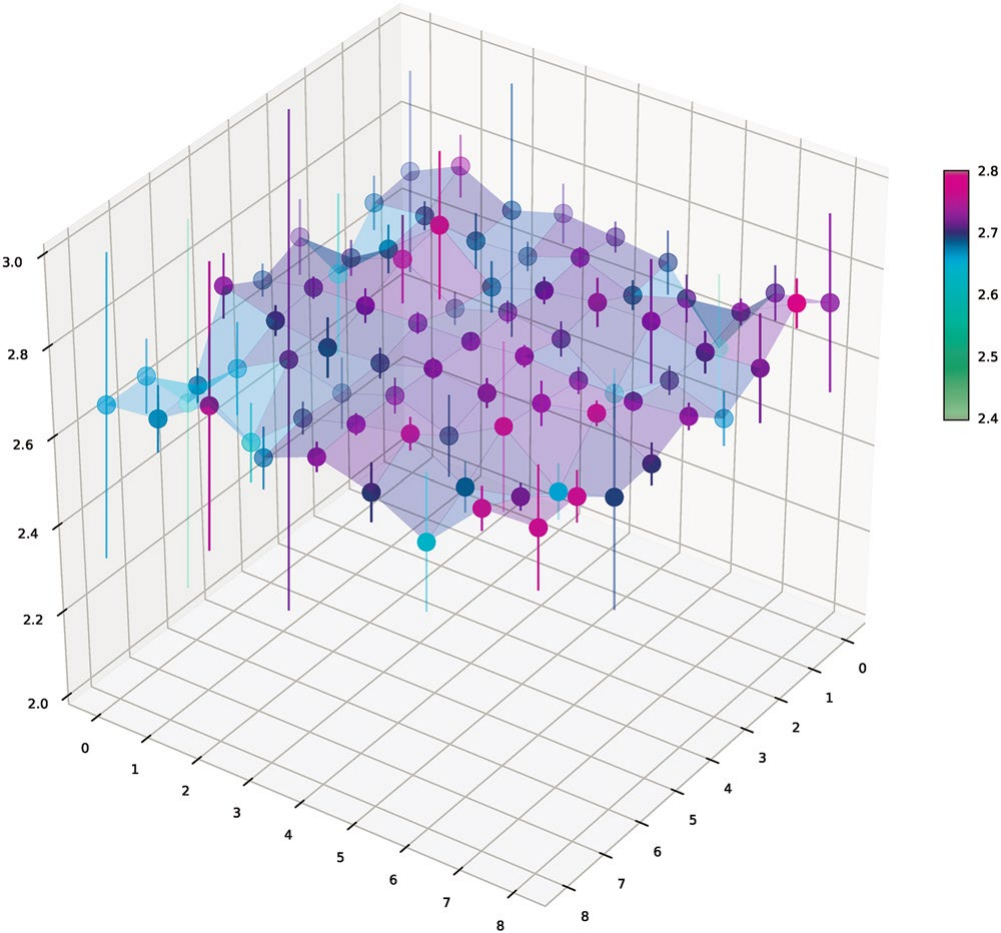
3D-representation of *S*-values for sub-area matrices with global *X* and *Y* indices as shown in Fig. 7.

One of the clear advantages of using high-speed cameras for quantum state characterization lies in the capacity to analyze multiple processes simultaneously. In our final experiment, we probe the ability of the fast camera to analyze 81 entangled pairs in parallel. To simulate the latter, we divided the areas illuminated by the fiber’s output into nine subareas, forming a 3 × 3 matrix, as shown in the two fiber regions in Fig. 3. Then, we analyze each pair-wise combination (81 total combinations) independently and reproduce the Bell analysis presented above for each of them. To accumulate enough statistics for these spatially resolved measurements, we took one-hour-long extended datasets, corresponding to the 16 combinations of the polarizer settings, which are needed to calculate the Bell’s inequality violation. The total number of recorded photons was considerable, about 10^9^, and required efforts to implement parallel processing of the data. The data analysis was performed within the scientific software framework *ROOT* developed at CERN^37^. Figure 7 shows the results of the parallel evaluation of 81 Bell’s inequalities. Each box represents a specific spatial combination of subareas. The corresponding S-value is color-coded, with the corresponding uncertainty shown in the center of the box. The results show that the S-value is uniform within the experimental errors with no apparent position dependence as expected. Figure 8 is a 3-dimensional representation of the same information as in Fig. 7.

## Discussion and Outlook

We have demonstrated that the spatial characterization of photonic entanglement can be performed employing the intensified Tpx3Cam camera. The camera can simultaneously time-stamp multiple single optical photons with nanosecond timing resolution while capturing their spatial information. The S-value results confirm that the fast camera spatial characterization of quantum-states in parallel is a viable alternative to be used in scaled-up quantum systems. The photon rate in these experiments was limited by the photon source to about 100 kHz. This is a factor of 100 lower than the maximum rate allowed by the camera, which should be capable of working with much brighter sources of entangled photons. Another specification of the fast camera, the Quantum Efficiency (QE) of the image intensifier, remains another critical parameter for the efficient detection of entangled photons. This technology is moving forward benefiting from new photocathodes based on GaAs with better QE, up to 35% in the same wavelength range as used for these studies^38^.

New imaging technologies based on monolithic silicon devices, such as single-photon avalanche devices (SPADs), are rapidly improving and could soon become competitive. Since the devices have internal amplification, the image intensifier is not necessary, which is a considerable simplification. Additionally, SPADs could have better time resolution and, potentially, higher QE, compared to the intensified cameras. The SPAD imagers started to appear on the market, and the first applications for quantum information science (QIS) have been reported^16,39–41^. Currently, the main limitation of the devices is the high dark count rate in the tens of MHz/cm^2^ range, which may saturate the readout and lead to low signal-to-background ratio. Another difficulty is the integration of the photon sensing SPAD pixels and sophisticated readout electronics in a monolithic device, which has many technical challenges. Also, in a SPAD, a single-photon fires only one pixel producing a standard pulse, so no centroiding is possible, therefore rendering it also impossible to distinguish a noise hit from a useful signal.

From the QIS perspective, we have showcased the possibility of parallel processing of tens of entangled states in parallel by analyzing independent combinations of subareas illuminated by the two fibers, which is an unprecedented capability for quantum information processing. As all pixels of the Tpx3Cam sensor act independently of each other, the dimensionality of this processing can be scaled up many-fold, for example, employing the same camera setup with brighter photon sources and/or multiple photon beams. We estimate that Tpx3Cam can successfully process at least 10 × 10 = 100 photon beams, each with a photon rate similar to the one used in these experiments. This technique may become a crucial tool for the real-time characterization of the performance for sizable entanglement-based quantum networks or circuits. Furthermore, the camera also can count the number of simultaneous photons in the same fiber, given sufficient spatial separation. This offers the possibility of discerning an event with more than one photon pair, an effect of the statistical distribution of the number of photons at the output of the SPDC process.

We envision that our characterization setup can prove useful in other quantum information processing tasks, such as Hong-Ou-Mandel interference^22^ and the characterization of entanglement encoded in orbital angular momentum (OAM) modes^42^. Furthermore, it is well suited for the real-time benchmarking of quantum memories using OAM states^43,44^, and for the parallel processing of the information in many memories systems^45^. Lastly, it could also find a niche as a feedback tool in the positioning of long-distance free-space quantum communication channels forming intra-city quantum cryptographic networks^46^.

## Data Availability

The data that support the findings of this study are available upon request.
